# 基于金属有机框架HKUST-1功能化聚合物整体柱的环境水样中痕量微囊藻毒素微萃取与分析

**DOI:** 10.3724/SP.J.1123.2026.01021

**Published:** 2026-11-08

**Authors:** Junying ZHENG, Hongyan ZENG, Xiaoping WU

**Affiliations:** 福州大学食品安全与生物分析教育部重点实验室，福州大学化学学院，福建 福州 350116; Key Laboratory for Analytical Science of Food Safety and Biology，College of Chemistry，Fuzhou University，Fuzhou 350116，China

**Keywords:** 微囊藻毒素, 金属有机框架, 功能化整体柱, 毛细管微萃取, 液相色谱-串联质谱, microcystins （MCs）, metal-organic frameworks （MOFs）, functionalized monolithic column, capillary microextraction （CME）, liquid chromatography-tandem mass spectrometry （LC-MS/MS）

## Abstract

微囊藻毒素（MCs）是一类环状七肽毒素，可造成水体污染，并能在水生生物体内富集、沿食物链传递，对人类健康构成严重威胁。然而，环境水样中复杂基质的干扰以及MCs的痕量存在，给其分析检测带来了严峻挑战，亟需开发高效的预处理技术加以克服。本研究利用离子液体介导反应快速聚合制备了一种金属有机框架HKUST-1功能化聚合物整体柱（HKUST-1@poly（DVB-co-AM-co-GMA）），并将其作为毛细管微萃取固定相用于富集水中的MCs。整体柱的制备是以甲基丙烯酸甘油酯（GMA）和丙烯酰胺（AM）为双功能单体，掺杂金属有机框架HKUST-1，以二乙烯苯（DVB）为交联剂，1-己基-3-甲基咪唑四氟硼酸盐（［HMIM］［BF_4_］）和环己醇为二元致孔剂，热引发聚合而成。表面形貌特征和元素组成表征结果表明，金属有机框架HKUST-1的引入显著增加了整体柱材料的介孔和微孔占比，并提高了比表面积，改善了传统有机聚合物整体柱比表面积小和易溶胀的缺陷，提高了材料对MCs的富集效率。在最优萃取条件下，将HKUST-1功能化整体柱作为毛细管微萃取（CME）的萃取相，与液相色谱-串联质谱（LC-MS/MS）联用，分析环境水样中的3种MCs异构体，检出限为0.28~0.60 ng/L，定量限为0.94~1.99 ng/L，回收率为82.2%~112.0%，相对标准偏差（RSD）不大于7.9%（*n*=3）。研究表明，借助HKUST-1功能化整体柱提供的特异性金属识别位点、氢键相互作用和*π-π*相互作用，该方法可高效富集并灵敏检测环境水体中的超痕量MCs，应用前景良好。

## Abstract (en)

Microcystins （MCs） are cyclic heptapeptide toxins that cause aquatic pollution. Through bioaccumulation，they can induce irreversible cellular and organ damage and significantly increase the risk of liver cancer，posing a serious threat to human health. However，interference from complex matrices in environmental water samples，as well as the trace concentrations of MCs，necessitates the development of efficient pretreatment techniques. Capillary microextraction （CME） is a miniaturized sample preparation technique derived from in-tube solid-phase microextraction （SPME）. By integrating extraction and concentration into a single step，CME offers distinct advantages，including low sample consumption，rapid equilibration，and high extraction efficiency. The performance of CME is critically dependent on the properties of its stationary phase，which governs analyte retention and selectivity. Traditional organic polymer monolithic columns are widely used due to their simple preparation and the availability of diverse functional monomers. Nevertheless，their fabrication process often suffers from issues such as particle agglomeration and heterogeneous pore distribution. These structural irregularities lead to limited specific surface area，poor mechanical stability，and ultimately，compromised extraction efficiency. With their high specific surface area，uniform pore size distribution，and highly tunable surface properties，metal-organic frameworks （MOFs） are ideal adsorbents that effectively overcome the limitations in porosity and specific surface area associated with traditional polymer-based materials. This study employed an ionic liquid-mediated ionothermal reaction to rapidly fabricate a metal-organic framework HKUST-1-functionalized polymer monolith column （HKUST-1@poly（DVB-co-AM-co-GMA）），which was used as a capillary microextraction （CME） medium to enrich MCs in water. In detail，the functionalized monolithic column was prepared using glycidyl methacrylate （GMA） and acrylamide （AM） as bifunctional monomers，divinylbenzene （DVB） as the crosslinking agent，a mixture of 1-hexyl-3-methylimidazolium tetrafluoroborate （［HMIM］［BF_4_］） and cyclohexanol as dual porogens，and HKUST-1 as the dopant. The morphology，elemental composition，specific surface area and pore size distribution of the monolithic material were characterized using scanning electron microscopy （SEM），Fourier transform infrared spectroscopy （FT-IR），X-ray photoelectron spectroscopy （XPS） and nitrogen adsorption–desorption isotherms. The results indicate that the incorporation of the metal-organic framework HKUST-1 significantly increases the proportion of mesopores and micropores in the monolithic column material. This enhancement increases the specific surface area and overcomes the low porosity，small surface area，and swelling susceptibility characteristic of traditional organic polymer monolithic columns. Consequently，the extraction efficiency of the proposed MOF hybrid monolith for enriching MCs is significantly improved. Meanwhile，the effects of extraction conditions （sample pH，sample flow rate） and elution conditions （formic acid concentration in eluent，elution flow rate） on enrichment efficiency were systematically investigated. Under optimized extraction conditions，the HKUST-1-functionalized monolith-based CME coupled with liquid chromatography-tandem mass spectrometry （LC-MS/MS） was employed to enrich and analyze three MC isomers in environmental water samples. Over the mass concentration range of 0.01-10 µg/L，the three MCs exhibited good linearity （*R*^2^≥0.995 5） between their mass concentrations and the peak areas of corresponding quantified ions. The limits of detection （LODs） and quantification （LOQs） were 0.28-0.60 ng/L and 0.94-1.99 ng/L，respectively，both significantly below the World Health Organization （WHO） maximum permissible concentration for MC-LR in water. The established analytical method yielded recoveries ranging from 82.2% to 112.0% for three environmental water samples，with relative standard deviations （RSDs）≤7.9% （*n*=3），exhibiting satisfactory precision and accuracy. Compared to other reported methods，the developed method requires less sample solvent （1.0 mL），achieves a lower LOD，and completes enrichment within 30 min. In conclusion，we demonstrate that the hyphenated analytical method efficiently detects ultra-trace MCs in environmental water，due to the specific coordination，hydrogen bond and *π-π* interactions afforded by the HKUST-1 functionalized monolith. This method shows promising value for monitoring trace environmental contaminants and advancing miniaturized pretreatment techniques.

微囊藻毒素（microcystins，MCs）是淡水和海水中大量繁殖的蓝藻代谢产物，也是水环境富营养化过程中最易于产生的结构性质稳定的藻类毒素之一^［1］^。由于其特殊的环状七肽结构，MCs在较宽的pH和温度范围内难以降解，因而在蓝藻水华过程中的产生和释放易引起严重的水体污染，威胁水生生态及人类健康。作为一种强烈的肝毒素，MCs能抑制蛋白磷酸酶1和蛋白磷酸酶2A的活性，提高肝癌的发病率^［2］^，并引发多器官慢性病变^［3］^。目前已报道的微囊藻毒素异构体有100多种^［4］^，其中，分布较广泛的MC-LR、MC-RR、MC-YR（L、R、Y分别代表亮氨酸、精氨酸和酪氨酸）常作为MCs分析的3项代表性指标^［5］^。在这3种毒素中，MC-LR因毒性最强且污染最为严重而研究最多，世界卫生组织（WHO）因此将饮用水中MC-LR的安全限量规定为1.0 µg/L^［6］^。由于蓝藻水华污染严重，我国海河流域部分支流中MC-LR的含量已高达µg/L水平^［7，8］^。因此，加强对环境水中痕量MCs的高灵敏检测对保障饮用水安全和人体健康十分必要。

目前MCs的检测方法主要包括酶联免疫吸附测定（ELISA）、高效液相色谱（HPLC）、液相色谱-串联质谱（LC-MS/MS）、高效毛细管电泳（HPCE）、生物传感器法等^［9-14］^，其中LC-MS/MS因其优异的选择性与灵敏度而被纳入多项国家标准，用于MCs的定性定量分析^［15］^。由于环境水样中MCs通常以痕量水平存在，复杂基质成分易产生离子抑制/共洗脱，对分离和定量产生干扰，色谱分析时采用直接进样通常无法达到所需灵敏度，必须依赖高效的样品预浓缩处理，以简化样品基质，保障后续分析的准确性。

固相微萃取技术（SPME）具有小型化、便携化、省时、绿色环保以及便于与分析仪器联用等特点，契合当今对萃取装置的需求^［16］^。在SPME基础上发展的毛细管微萃取（CME）是一种以熔融石英毛细管为载体、在内壁上以物理或化学键合方式涂覆材料的微型管内SPME装置，具有样品消耗少、平衡时间短、萃取效率高的特点。CME的固定相是影响其萃取性能的关键因素^［17，18］^。有机聚合物整体柱制备简单、单体选择多样、pH范围广、表面易改性，特别适用于生物大分子的分离富集^［19］^。然而，传统聚合物整体柱在制备过程中易出现颗粒堆积和孔道不均匀的问题，导致其稳定性差和比表面积有限，进而影响整体材料的萃取效率。此外，通常还需在整体柱表面进行功能基修饰，才能满足对目标物的富集要求。因此，开发有序性好、稳定性高且兼具高孔隙率与高比表面积的功能化整体材料，是实现MCs高效萃取富集的关键。

金属有机框架材料（MOFs）是一种由金属离子或离子簇与有机配体通过配位键连接而成的有序晶状多孔材料^［20］^，组成和结构极为丰富。由于具有高比表面积、均匀的孔径分布和高度可调的表面性质，MOFs成为一类理想的吸附剂，可弥补传统的聚合物基吸附剂在孔隙率和比表面积方面的不足^［21］^。近年来，利用MOFs或其纳米复合材料富集MCs的研究已取得一系列进展，其中磁性MOFs复合材料^［22，23］^、MOFs改性SPME纤维^［24］^以及离子液体辅助的MOFs原位生长策略^［25］^等新技术的出现，证实了MOFs在毒素富集分析领域的应用潜力。

本研究根据MCs的结构特点，引入金属有机框架HKUST-1，以甲基丙烯酸甘油酯（GMA）和丙烯酰胺（AM）为双功能单体，二乙烯苯为交联剂，1-己基-3-甲基咪唑四氟硼酸盐（［HMIM］［BF_4_］）和环己醇为二元致孔剂，通过离子液体介导的离子热快速聚合反应，制备了HKUST-1@poly（DVB-co-AM-co-GMA）功能化聚合物整体柱，采用扫描电子显微镜（SEM）、傅里叶变换红外光谱（FT-IR）、氮气吸附-脱附等温测试等表征手段对整体材料的形貌特征、元素组成、比表面积以及孔径分布进行探究。基于MOFs大比表面积、多孔、识别位点丰富的特性，在聚合物基质上修饰HKUST-1框架可以为MCs分子提供不饱和金属位点、*π-π*相互作用以及氢键相互作用，协同增强对MCs的吸附性能。通过将基于HKUST-1功能化整体柱的CME与LC-MS/MS技术联用，成功建立了一种能够高效富集、高灵敏检测水中痕量MCs的方法，为环境监测和水污染控制提供了新工具。

## 1 实验部分

### 1.1 仪器与试剂

LCMS-8040液相色谱-质谱联用仪（日本Shimadzu公司）；NE-1002X注射泵（美国New Era公司）；LC-10ADvp高压恒流泵（日本Shimadzu公司）；Thermo Nicolet 6700 傅里叶变换红外光谱仪（FT-IR，美国Thermo Fisher公司）；ASAP 2460比表面积及孔径分析仪（BET，美国Micromeritics公司）；Zetasizer 3000HSA纳米粒度及电位分析仪（英国Malvern公司）；Nova Nano SEM 230场发射扫描电子显微镜（SEM，美国FEI公司）；ESCALAB 250 X射线光电子能谱仪（XPS，美国Thermo Fisher公司）。

AM、GMA、二甲基亚砜（DMSO）、1，3，5-均苯三甲酸（H_3_BTC）、环己醇购自阿拉丁试剂有限公司（上海）。［HMIM］［BF_4_］购自上海成捷化学有限公司（上海）。二乙烯苯（DVB）、偶氮二异丁腈（AIBN）购自麦克林生化科技有限公司（上海）。三水合硝酸铜（Cu（NO_3_）_2_·3H_2_O）购自上海沪试实验室器材有限公司。甲醇（色谱级）、乙腈（ACN，色谱级）、无水乙醇（色谱级）、甲酸（FA，色谱级）、氨水、盐酸和氢氧化钠购自国药化学试剂有限公司（上海，中国）。微囊藻毒素MC-LR、MC-RR、MC-YR标准品（纯度>98%）购自Alexis生物公司（美国）。除标注外，其他实验试剂均为分析纯。实验用超纯水（18.2 MΩ·cm）由美国Milli-Q净水系统（Merck Millipore，德国）制备，实验用石英毛细管（320 µm i.d.× 450 µm o.d.）购自河北永年光导纤维厂。0.22 µm尼龙滤膜购自上海兴亚净化材料厂。

MCs标准储备液：用1 000 µL甲醇溶解1.0 mg的MCs标准品（MC-LR、MC-RR和MC-YR），各配成1.0 mg/mL的MCs标准储备液。将储备液置于2 mL离心管内存放于-20 ℃冰箱中，需要使用时室温解冻涡旋均匀后，用超纯水稀释至所需浓度。

### 1.2 HKUST-1的合成及HKUST-1@poly（DVB-co-AM-co-GMA）整体柱的制备

#### 1.2.1 HKUST-1的合成

HKUST-1晶体的合成在室温合成法^［26］^的基础上进行了改进：将1.22 g Cu（NO_3_）_2_·3H_2_O和0.58 g H_3_BTC溶解在5 g DMSO中，之后对混合液进行涡旋、超声处理以确保溶质完全分散，得到HKUST-1的前驱液。在前驱液中加入50 mL甲醇，室温搅拌24 h以充分反应得到蓝色悬浊液，离心分离后，将沉淀物用甲醇洗涤3次除去未反应杂质，于80 ℃烘箱中干燥12 h，得到蓝色的HKUST-1晶体粉末。

#### 1.2.2 HKUST-1功能化整体柱的制备

在制备功能化整体柱之前，首先参照前期工作^［27］^对石英毛细管内壁进行烯基化处理，接着利用离子热合成法“一锅”制备了HKUST-1@poly（DVB-co-AM-co-GMA）功能化整体柱，具体制备流程见图1。以AM（16 mg）、GMA（24 mg）为双功能单体，DVB（60 mg）为交联剂，［HMIM］［BF_4_］（60 mg）和环己醇（40 mg）为二元致孔体系，AIBN（0.4 mg）为引发剂，同时添加5 mg的HKUST-1，构成预聚合液，涡旋30 s使其充分混合，超声10 min除去气泡。用玻璃注射器将处理后的预聚合液灌入内壁烯基化的毛细管柱中，两端用硅胶塞封住，于95 ℃的水浴中快速反应10 min后取出。用甲醇冲洗整体柱30 min，除去可能存在的杂质和未反应的致孔剂，最终得到HKUST-1@poly（DVB-co-AM-co-GMA）功能化聚合物整体柱。

**图1:**
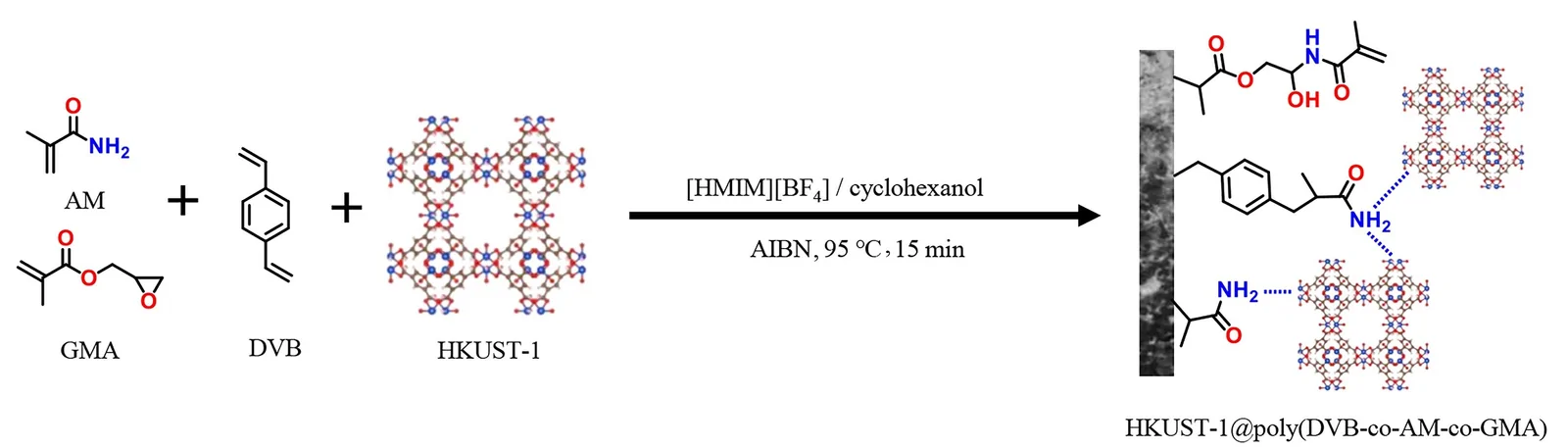
HKUST-1@poly（DVB-co-AM-co-GMA）功能化整体柱的制备流程图 AM： acrylamide； GMA： glycidyl methacrylate； DVB： divinylbenzene； AIBN： 2，2′-azobisisobutyronitrile.

### 1.3 毛细管微萃取富集过程

CME富集的装置包括注射泵、鲁尔接头、聚醚醚酮（PEEK）接头、微量注射器。截取3 cm整体柱，在富集前先用甲醇活化30 min；之后将1 mL MCs标准溶液或者样品溶液装入微量注射器中，将整体柱用PEEK头和鲁尔接头与微量注射器连接。以25 µL/min的流速，将上样液泵入整体柱中；待吸附完成之后，先用超纯水冲洗整体柱以除去未结合的目标物，然后以甲醇-2%甲酸水溶液（80∶20，体积比）为洗脱液，以45 µL/min的解吸流速将目标物洗脱，收集的洗脱液经过0.22 µm尼龙滤膜过滤后进行LC-MS/MS分析。

采用测量压力变化的方法计算渗透性：将截取的有效柱长5 cm的整体柱连接在液相色谱泵上，使用纯甲醇为流动相进行压力实验，流速0.05 mL/min，记录柱压力稳定时的压力差Δ*P*。根据文献［^28^］的方法，按式（1）对整体柱的渗透性进行计算。


$$Κ=F\eta L/\left(\pi R^{2}\Delta P\right)$$ (1)

其中，渗透率*K*（m^2^）的计算通常基于达西定律（Darcy’s law），具体涉及以下参数：柱压降Δ*P*（Pa）、有效柱长*L*（cm）、流动相的动力学黏度*η*（Pa·s）（甲醇的动力学黏度为0.544 mPa·s）、流速*F*（m^3^/s）、毛细管内径*R*（m）。

通过动态前沿分析法测定MCs在HKUST-1功能化聚合物整体柱上的穿透曲线，结合目标物的流速和质量浓度计算最大负载量，见式（2）。


$$Q_{max}=C\left(V_{R}-V_{0}\right)/m$$ （2）

其中，*Q*_max_为整体柱对目标物的最大负载量（μg/mg）；*C*为目标物标准溶液的质量浓度（μg/L）；*V*_R_为目标物在功能化整体柱上的保留体积（μL）；*V*_0_表示功能化整体柱的死体积（μL）；*m*代表整体柱内材料的质量（mg）。根据文献［^29^］的方法，将穿透曲线最大响应值中间点的体积定为保留体积*V*_R_。

### 1.4 LC-MS/MS分析方法

用LC-MS/MS对经CME富集后的微囊藻毒素进行定量分析。

色谱条件 Shim-pack GIST C18色谱柱（150 mm×2.1 mm，5 µm）。流动相A为含0.1%FA的乙腈溶液，流动相B为含0.1%FA的水溶液；在常温下设置梯度洗脱程序，在0~4 min内，A相比例由30%均匀升高至70%（体积分数），4~7 min内均匀降低A相的比例（从70%降低至30%），7~8 min维持A相比例为30%，单次进样流程总用时8 min；进样体积为10 µL。

质谱条件 离子源：电喷雾电离源（ESI）；扫描方式：正离子模式；检测方式：多反应监测（MRM）模式；喷雾电压：4 500 V；脱溶剂温度：250 ℃；干燥气温度：400 ℃；雾化气：N_2_；雾化气流量：3.0 L/min；雾化气压力：0.97 MPa；碰撞气体：Ar。表1为3种MCs的MS/MS参数。

**表1:** 3种MCs的MS/MS参数

Analyte	Precursor ion （*m/z*）	Product ions （*m/z*）	Collision energies/eV
MC-RR	519.9	135.1^*^，127.1	36，22
MC-LR^a^	498.4	135.1^*^，861.5	14，11
MC-YR^a^	523.4	135.1^*^，911.5	19，9

a. doubly charged MC. * Quantitative ion.

### 1.5 样品预处理

河水样品取自福州市的晋安河，水库水样取自福州市晋安区的登云水库，湖水样品取自福州大学旗山校区国家大学科技园的湖泊。所有样品储存在4 ℃冰箱内。水样使用0.22 µm尼龙滤膜过滤后直接用于MCs检测。在环境水样中添加不同水平的MCs混合标准溶液（0.05、0.50、5.0 µg/L，包括MC-RR、MC-LR和MC-YR），制备一系列加标水样用于回收试验。

## 2 结果与讨论

### 2.1 整体柱的制备条件考察

本研究利用离子热合成法，在毛细管整体柱中一锅法制备得到HKUST-1@poly（DVB-co-AM-co-GMA）功能化整体柱。以1 µg/L MC-LR的色谱峰面积作为整体柱对目标物萃取效率的判定指标，并综合柱渗透率、稳定性与MC-LR富集效果等因素，对制备整体柱的预聚合液中的单体比例（双功能单体、HKUST-1）、单体与交联剂、致孔剂比例等多种条件进行优化（表2）。两种功能单体（GMA、AM）通过烯基与交联剂相连形成整体材料，结果表明，降低GMA/AM含量比会使整体材料的结构变得更致密、渗透率降低（柱A和柱C），然而该比例下整体材料的机械强度或与柱管壁的结合力不足，使其在上样压力波动时易被冲出，进而影响柱结构的稳定性。而AM含量的减少则会导致柱表面功能基团减少，降低对MC-LR的富集效果（如柱A和柱B），因此选取GMA∶AM=24 mg∶16 mg为最优功能单体比例。调整良溶剂［HMIM］［BF_4_］与非良溶剂环己醇的比例，可以有效调节整体柱的形貌和孔的尺寸，进而影响其通透性和富集效果。提升良溶剂［HMIM］［BF_4_］比例（如柱A和柱D），整体柱倾向于生成更小的孔，大孔比例降低、渗透率下降，但小孔可提供更多的吸附表面积，富集效果提升；降低良溶剂比例（如柱A和柱E），柱通透性与传质能力增强、富集效率提高，但材料与内壁结合性变差，易在上样洗脱时冲出；综合考量，最终选取［HMIM］［BF_4_］∶环己醇=60 mg∶40 mg为最佳致孔剂比例。适量添加HKUST-1可提升整体柱材料的比表面积，为富集过程提供金属离子络合作用以及苯环、羧基等功能基，但过量添加会堵塞孔道导致富集效率下降。综合考虑渗透率与富集效率，最后选择柱H用于后续MCs的富集。

**表2:** HKUST-1@poly（DVB-co-AM-co-GMA）功能化整体柱的预聚合液组成

Column	HKUST-1/mg	GMA/mg	AM/mg	DVB/mg	［HMIM］［BF_4_］/mg	Cyclohexanol/mg	Permeability （*K*）/（10^-14^ m^2^）	*S*_MC-LR_
A	0	24	16	60	60	40	15.3	207959
B	0	26	14	60	60	40	15.3	150628
C	0	22	18	60	60	40	7.66	171502
D	0	24	16	60	65	35	7.66	208332
E	0	24	16	60	55	45	15.3	219714
F	1	24	16	60	60	40	15.3	233172
G	3	24	16	60	60	40	7.66	240598
H	5	24	16	60	60	40	7.66	254036
I	7	24	16	60	60	40	3.83	198018

*S*_MC-LR_： peak area of MC-LR.

### 2.2 整体柱的结构表征

通过扫描电镜观察HKUST-1功能化整体柱的表面形态和内部结构，如图2a、2b所示，整体材料与毛细管内壁紧密结合，未出现脱壁现象；如图2c所示，整体材料呈现颗粒较小且致密的花椰菜状，颗粒表面暴露有密集微小八面体状的HKUST-1晶体；这些晶体的存在不仅增加了整体材料的比表面积，还为吸附提供了更多的活性位点，从而提升整体材料对目标物的富集效率。颗粒之间分布着较大的通孔，确保了柱子的通透性，这对于色谱分离和富集过程中的传质至关重要。

**图2:**
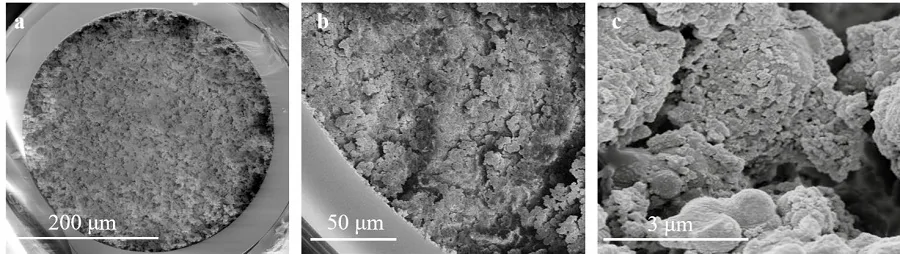
HKUST-1@poly（DVB-co-AM-co-GMA）整体柱的扫描电镜图

如图3所示，对比HKUST-1@poly（DVB-co-AM-co-GMA）整体柱与AM、DVB、GMA和HKUST-1的红外吸收光谱图，HKUST-1@poly（DVB-co-AM-co-GMA）在1 113 cm^-1^处出现AM谱中酰胺基C-N的伸缩振动峰，在1 727 cm^-1^处出现GMA谱中C=O的伸缩振动峰，在836、798、708 cm^-1^处出现DVB谱中=C-H的面外弯曲振动和苯环C-H面外弯曲振动，在1 449 cm^-1^和1 378 cm^-1^处出现HKUST-1谱中COO^-^的对称伸缩振动，进一步证明了整体柱的成功合成。

**图3:**
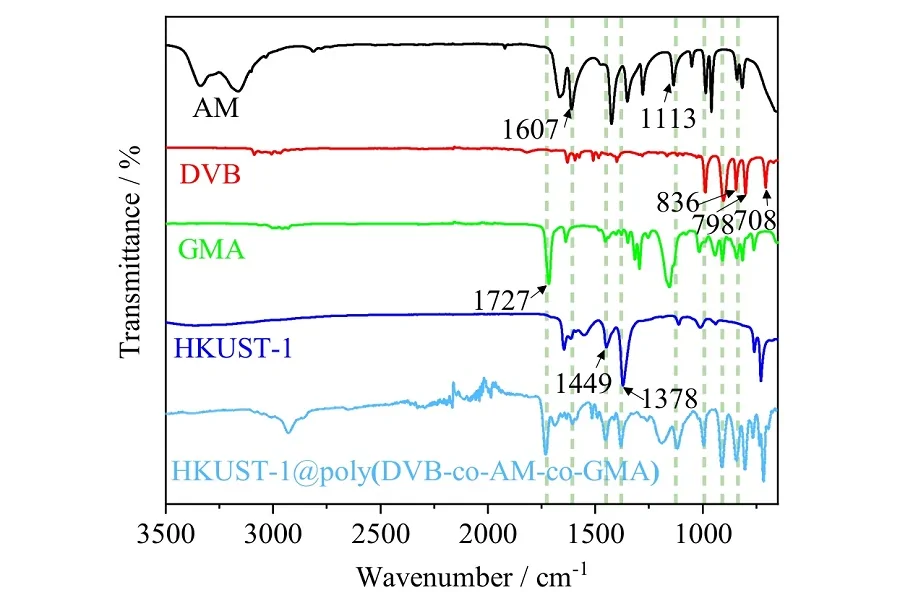
AM、DVB、GMA、HKUST-1及HKUST-1@poly（DVB-co-AM-co-GMA）功能化整体柱的FT-IR谱图

HKUST-1@poly（DVB-co-AM-co-GMA）功能化整体材料的氮气吸附-脱附等温曲线如图4所示，呈现出Ⅲ型等温线，比表面积为11.97 m^2^/g，平均孔径为7.44 nm，具有介孔和微孔结构。结果表明掺杂HKUST-1后，整体材料依旧能保持良好的通透性，且具备致密性和较大的比表面积，这为其优异的富集性能提供了保障。

**图4:**
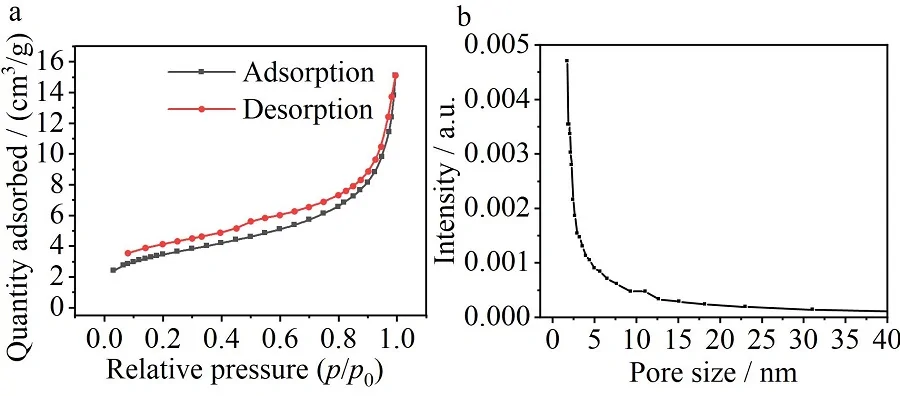
HKUST-1@poly（DVB-co-AM-co-GMA）功能化整体材料的（a）氮气吸附-脱附等温曲线和（b）孔径分布图

### 2.3 CME萃取条件优化

利用HKUST-1@poly（DVB-co-AM-co-GMA）整体柱进行MCs的CME萃取，并联用LC-MS/MS分析。为了获得最佳的富集效率，我们通过单因素实验考察了上样液pH、上样流速、洗脱液FA含量、洗脱流速等影响因素。以1.0 µg/L MCs（包括MC-LR、MC-YR和MC-RR）的色谱峰面积作为参考指标，通过实验优化得出最优的萃取条件（见图5）。

**图5:**
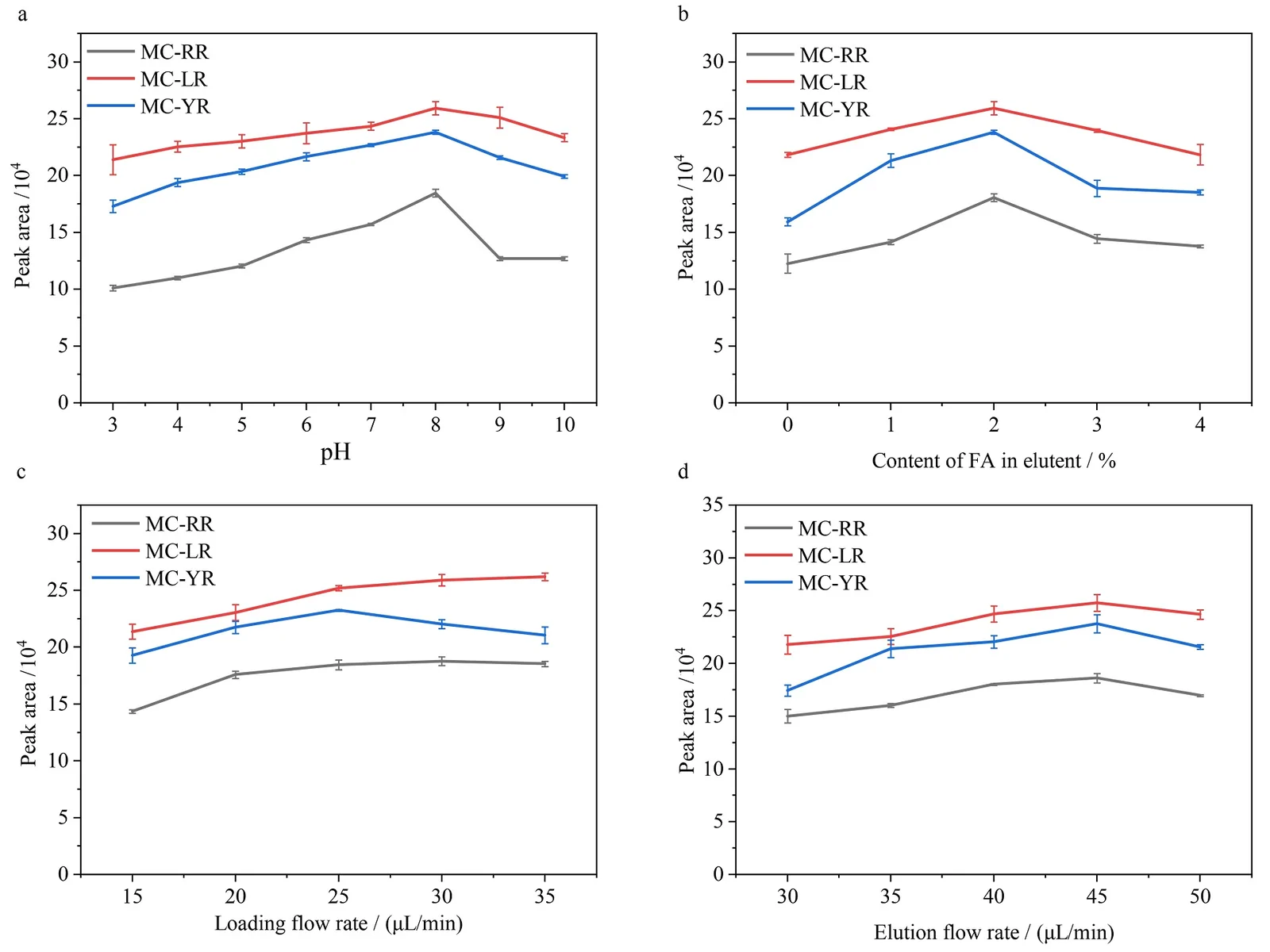
（a）上样液pH、（b）洗脱液中FA的含量、（c）上样流速和（d）洗脱流速对3种MCs萃取效率的影响（*n*=3）

#### 2.3.1 上样液pH

pH对MCs的存在形式和整体柱表面的带电情况有显著影响，是影响富集效率的关键因素。实验探究了上样液pH为3.0~10.0时MCs富集效率的影响，如图5a所示，3种MCs的峰面积响应值随着pH增大而逐渐增加并在pH=8.0时达到最大。这是由于逐渐增加的pH使得微囊藻毒素分子结构中氨基和羧基的解离程度增大（3种MCs的p*K*_a_值为3.3~3.4^［30］^），而MCs释放出的H^+^增强了其与整体柱之间的氢键相互作用，提高了在整体柱上的吸附能力。然而，pH大于8后，MCs峰面积明显下降。这种峰面积的降低通常是多种作用力协同产生的，如疏水、静电和*π-π*相互作用^［31］^；此时，MCs表面羧基全部解离并且呈现出更大的负电性，MCs分子更倾向于保留在水相中，而不是吸附在吸附剂上。所以，目标物富集效果显著降低，特别是结构中带两个胍基的MC-RR，在pH=9的时候与整体材料表面产生很大的静电排斥，响应值显著下降。基于以上实验结果，选择pH=8.0的上样液进行后续实验。

#### 2.3.2 洗脱液组成

由于整体柱材料具有不饱和金属位点（Cu^2+^），加入FA可能在材料表面与MCs形成竞争吸附，从而减弱了材料与MCs中羧基和氨基的配位作用，将利于MCs的洗脱。为了保证目标物能够被充分洗脱，实验考察了含有不同FA含量的洗脱液对富集效率的影响，结果如图5b所示。随着洗脱液中FA含量从0增加到2%，MCs的响应值逐渐上升并达到最佳；然而，继续增加FA的含量反而使得3种MCs的峰面积有一定程度的降低，这可能是因为洗脱液中甲酸占比过高会使体系pH降至MCs的等电点附近，净电荷趋近于零，其水溶性下降^［32］^，同时过酸的环境导致MCs分子过度质子化，可能抑制MCs在质谱检测中的离子化效率，降低目标物的电离效率^［33］^，导致MCs的洗脱效率下降。这表明，适当增加FA含量能提高MC-LR的洗脱效率，但过高的FA含量则会对MCs分子的电离过程产生离子抑制效应，从而影响洗脱效率。因此，在后续的条件优化实验中，选择MeOH-2% FA水溶液（80∶20，体积比）的洗脱液组成。

#### 2.3.3 上样流速

上样流速对MCs在固定相的传质过程起着关键作用。在15~35 µL/min范围内考察了上样流速与峰面积之间的关系，结果如图5c所示，当上样流速从15 µL/min增加到25 µL/min时，目标物峰面积呈现上升趋势，之后趋于稳定甚至有所下降，这是因为高流速条件下目标物与吸附剂的接触时间减少，使得负载量变小。综合考虑，选定25 µL/min为后续优化实验的上样流速。

#### 2.3.4 洗脱流速

如图5d所示，随着洗脱流速的增加，整体柱对MCs的富集效率略有提升，并在洗脱流速为45 µL/min时达到最大。但进一步增加流速会降低洗脱液与吸附剂的接触时间，导致部分吸附的MCs来不及被完全洗脱。综合萃取时间和富集效果等因素，选择45 µL/min作为后续实验的洗脱流速。

综上所述，最优的MCs富集条件如下：样品溶液pH=8.0，洗脱液组成为（MeOH∶2% FA水溶液=80∶20，体积比），上样流速为25 µL/min，洗脱流速为45 µL/min。

### 2.4 整体柱的萃取机理

#### 2.4.1 整体柱的富集容量

为了评估HKUST-1@poly（DVB-co-AM-co-GMA）功能化整体柱对MC-LR的吸附容量，在最优萃取条件下，将配制的500 µg/L MC-LR的上样液以30 µL/min的流速泵入整体柱中。同时在尾端接收一定体积的流出液进行LC-MS/MS进样分析。通过以流出体积为横坐标，峰面积为纵坐标绘制穿透曲线。如图6所示，根据穿透曲线斜率中间点对应的*V*_R_，确定了HKUST-1@poly（DVB-co-AM-co-GMA）整体柱的*V*_R_=4 400 µL。根据式（2），计算出HKUST-1@poly（DVB-co-AM-co-GMA）功能化整体柱对MC-LR的最大负载量为2.68 μg/mg。

**图6:**
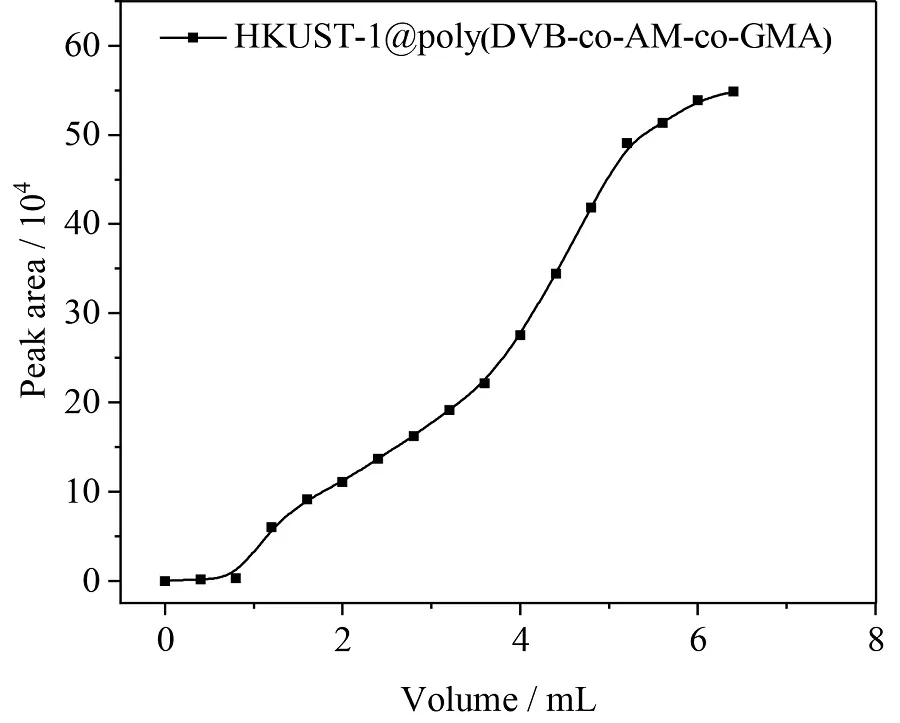
HKUST-1@poly（DVB-co-AM-co-GMA）整体柱对MC-LR的穿透曲线

#### 2.4.2 XPS光电子能谱

为了探究HKUST-1@poly（DVB-co-AM-co-GMA）功能化整体柱与MC-LR的吸附机理，本研究对比了整体柱结合MC-LR前后的XPS光谱（见图7）。通过分析表面结合能和化学键的变化，推测整体材料表面与MC-LR之间存在的相互作用力。

**图7:**
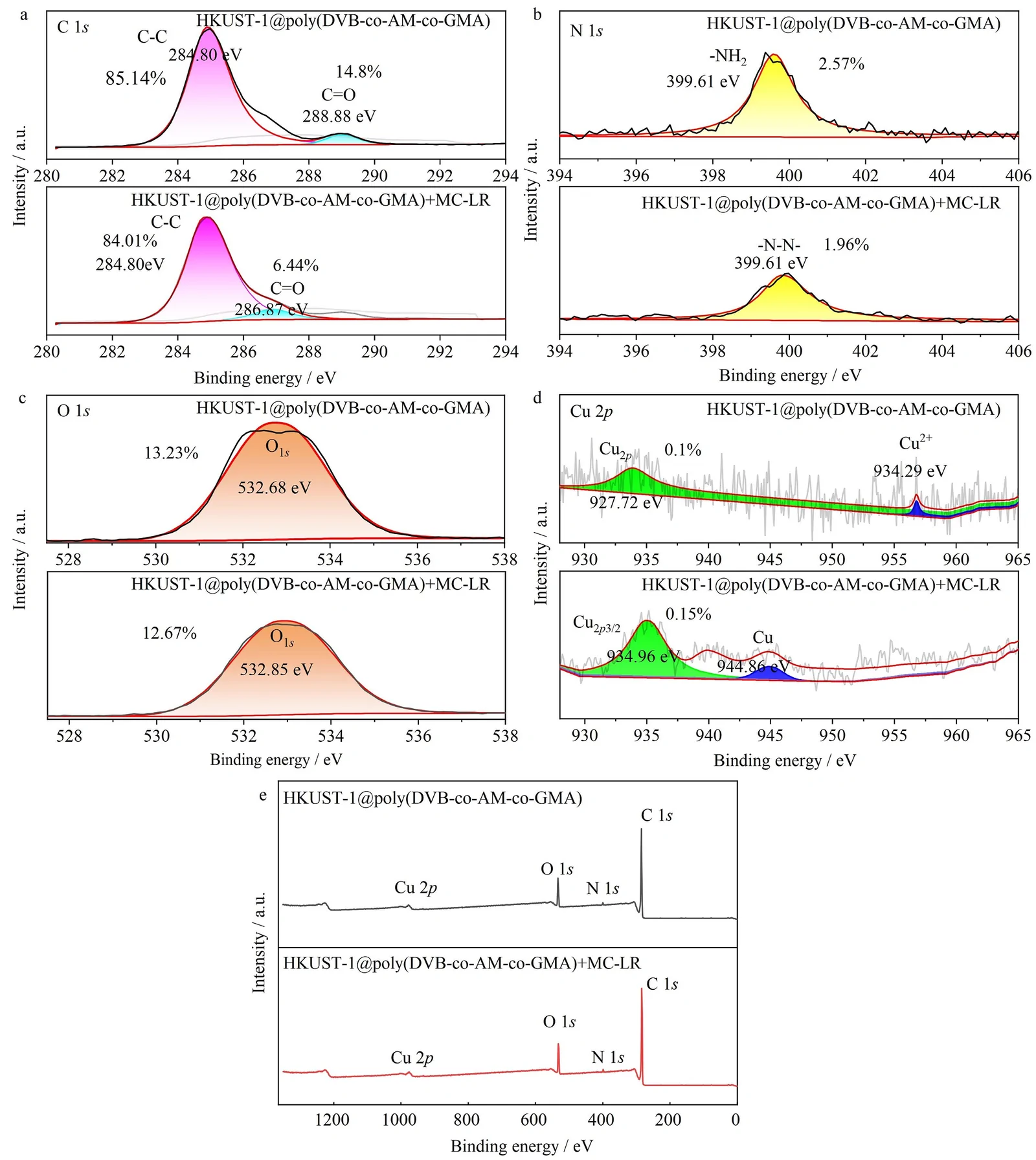
HKUST-1@poly（DVB-co-AM-co-GMA）整体柱结合MC-LR前后的（a） C 1*s*的XPS谱图、（b） N 1*s*的XPS谱图、（c） O 1*s*的XPS谱图、（d） Cu 2*p*的XPS谱图和（e） XPS全谱

根据图7e所示的XPS全谱图，可以清晰地观察到整体柱表面分布C、N、O、Cu元素，进一步验证了HKUST-1功能化整体柱的成功制备。图7a的C 1*s*光谱通过分峰拟合为2个峰，分别对应C-C/C=C、C=O。当整体柱吸附MC-LR后，C-C/C=C和C=O相对强度分别从原先的85.14%和14.8%降低至84.01%和6.44%，这一变化可能源于整体柱结构中的苯环与MCs中的苯环发生了*π-π*相互作用，从而改变了表面的电子分布状态。C=O的结合能从288.88 eV移动到286.87 eV，出现显著位移，常归因于碳原子周围电子云密度的增加，这可能源于整体柱表面的羰基氧原子参与了与MC-LR分子中极性基团（如羧基和氨基）的氢键相互作用，导致化学环境发生变化。同时，N 1*s*光谱中-NH_2_和O 1*s*光谱中O的相对含量出现下降（图7b、7c），进一步佐证了整体柱表面的氨基和含氧官能团在吸附过程中充当了活性位点，从而改变了结合能环境。在Cu 2*p*光谱（图7d）中，吸附MC-LR后，Cu^2+^的特征峰几乎完全消失，表明HKUST-1与MCs之间存在金属配位作用^［34，35］^。

XPS分析结果表明，HKUST-1@poly（DVB-co-AM-co-GMA）整体柱吸附MC-LR是多种机制协同作用的结果，主要作用力为HKUST-1中Cu^2+^与MC-LR的羧基之间的配位作用、柱结构中苯环与MC-LR的苯环之间的*π-π*相互作用以及整体柱表面的氨基与MC-LR的氨基/羧基之间的氢键作用。

### 2.5 方法学考察

在最佳实验条件下，将HKUST-1@poly（DVB-co-AM-co-GMA）功能化整体柱用于CME，并与LC-MS/MS联用，建立了环境水中3种MCs异构体的检测方法。3种MCs的质量浓度在0.01~10 µg/L范围内与定量离子峰面积具有良好的线性关系（表3）。方法的检出限（LOD，*S/N*=3）为0.28~0.60 ng/L，定量限（LOQ，*S/N*=10）为0.94~1.99 ng/L，远低于WHO规定的水中MC-LR的限量标准，说明所建立的方法具有较好的灵敏度。

**表3:** CME与LC-MS/MS联用方法对MCs的分析性能

Analyte	Linear range/（μg/L）	*R* ^2^	LOD/（ng/L，*S/N=*3）	LOQ/（ng/L，*S/N*=10）	RSDs/% （*n*=5）		
					Column-to-column	Inter-day	Intra-day
MC-LR	0.01-10	0.9955	0.28	0.94	3.0	1.4	1.4
MC-RR	0.01-10	0.9986	0.60	1.99	2.6	2.3	3.4
MC-YR	0.01-10	0.9995	0.48	1.60	3.5	3.7	2.6

为了验证方法的稳定性和重现性，在最优CME条件下，使用HKUST-1@poly（DVB-co-AM-co-GMA）功能化聚合物整体柱对1.0 µg/L MCs进行萃取，分别开展柱间、日内和日间重复性试验，计算MCs峰面积的相对标准偏差（RSD），结果见表3。可以看出，选用同一批次制备的5根功能化整体柱在相同条件下萃取MCs并测定，得到峰面积的RSD≤3.5%（*n*=5）；使用同一根功能化整体柱在同一天内和连续5天内对MCs进行CME-LC-MS分析（*n*=5），计算得到RSD≤3.7%。

此外，还考察了所制备整体材料的耐用性。在相同实验条件下，将同一根功能化整体柱连续重复使用10次，所得峰面积的RSD仍保持在3.2%以内（*n*=10）。在连续使用两个月后，该功能化整体柱富集MC-LR峰面积的RSD为5.7%（图8），这些结果充分证明了功能化整体柱良好的稳定性。

**图8:**
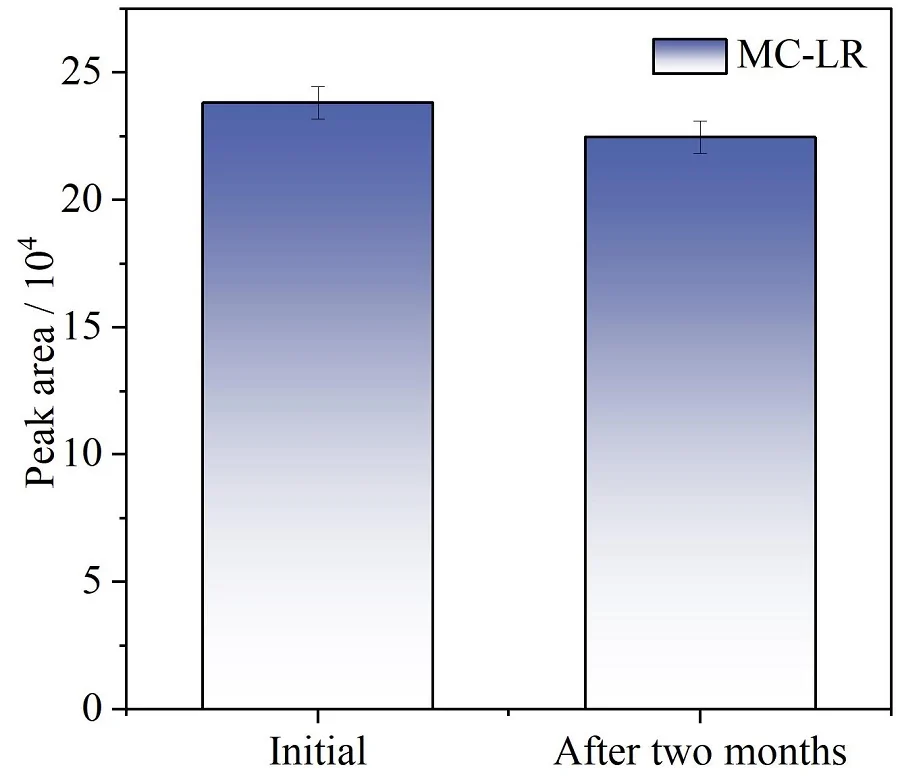
HKUST-1@poly（DVB-co-AM-co-GMA）功能化整体柱的重复使用性（*n*=3）

### 2.6 环境水样分析

利用所制备功能化整体柱对来自河水、湖水和水库水的实际水样中的痕量MCs进行CME富集，并联用LC-MS/MS检测。结果如图9所示，可以看出未经CME富集的空白水样（图9曲线a）几乎没有明显响应，而经过CME富集的水样（图9曲线b）则显示出增大的色谱峰。经CME富集后，我们在部分水库水、河水和湖水中检测到极其微量的MCs，其中MC-LR含量未超过国家饮用水标准的限值。这一结果证实了HKUST-1@poly（DVB-co-AM-co-GMA）功能化整体柱对3种MCs具有良好的富集能力，能够用于实际环境水样中MCs的同时检测。

**图9:**
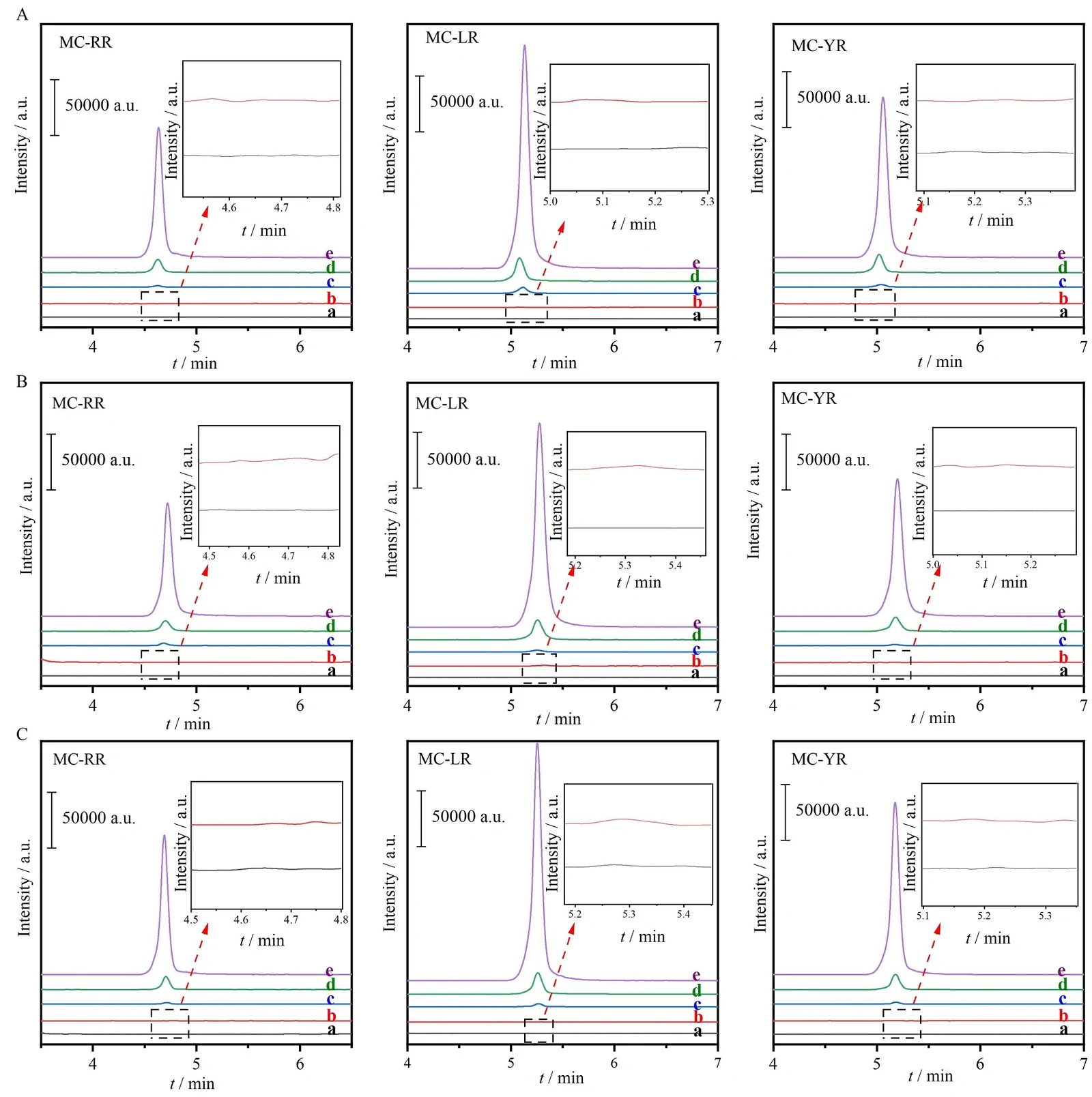
（A）水库水、（B）河水和（C）湖水样品中MCs的TIC谱图 a. direct analysis； b. sample analysis after CME enrichment； c. sample spiked with 0.05 µg/L MCs； d. sample spiked with 0.5 µg/L MCs； e. sample spiked with 5 µg/L MCs.

为了验证建立方法的准确性，本研究对实际水样进行了加标试验，并利用建立的CME与LC-MS/MS联用技术进行了回收率测试，结果见图9中曲线c、d、e及表4。结果表明，该方法展现出令人满意的MCs回收率（82.2%~112.0%）和精密度（RSD≤7.9%），表明环境水样中痕量MCs的CME萃取联用LC-MS/MS分析具有良好的准确性和精密度。

**表4:** CME与LC-MS/MS方法联用分析3种实际水样中MCs的回收率和精密度（*n*=3）

Sample	Spiked/（μg/L）	MC-LR			MC-RR			MC-YR	
		Detected/（μg/L）	Recovery （RSD）/%		Detected/（μg/L）	Recovery （RSD）/%		Detected/（μg/L）	Recovery （RSD）/%
Reservoir water	0	0.0027	/		N.D.	/		N.D.	/
	0.05	0.041	82.2 （5.5）		0.044	88.8 （3.0）		0.056	111.0 （7.9）
	0.50	0.53	105.0 （2.5）		0.56	112.0 （1.8）		0.49	98.5 （1.7）
	5.0	5.5	111.1 （7.0）		4.7	94.3 （2.3）		5.2	103.0 （4.2）
River water	0	0.0014	/		0.0023	/		0.0019	/
	0.05	0.045	90.5 （4.3）		0.052	104.0 （6.1）		0.057	101.0 （1.4）
	0.50	0.55	109.0 （4.3）		0.42	83.6 （5.8）		0.45	90.9 （3.0）
	5.0	5.0	101.0 （3.7）		4.6	93.2 （1.6）		5.1	101.0 （2.4）
Lake water	0	0.0012	/		0.0009	/		N.D.	/
	0.05	0.048	95.3 （4.3）		0.049	97.2 （4.7）		0.50	100.0 （2.6）
	0.50	0.51	102.0 （2.8）		0.42	83.2 （6.3）		0.49	99.1 （2.0）
	5.0	4.8	96.4 （6.0）		4.7	93.8 （1.7）		5.2	104.0 （6.3）

N.D.： not detected.

### 2.7 方法对比

与其他已报道的方法相比（表5），本研究建立的基于HKUST-1@poly（DVB-co-AM-co-GMA）整体柱的CME-LC-MS/MS方法所需样品溶液体积较小（仅需1.0 mL），且具有较低的检出限，能够在较短的时间（小于30 min）内完成富集过程。可见，该分析方法具有较快的富集速率和较高的灵敏度，适用于环境水样中MCs的检测分析。

**表 5:** 本方法与其他已报道的MCs分析方法的比较

Method	Adsorbent	Samples	Sample consumption/mL	LODs/（ng/L）	Recoveries/%	Ref.
MSPE-LC-MS/MS	DS-MMIP@GO	tap water，river water	1000	0.03-0.61	84.1-98.2	［^36^］
MSPE-HPLC-MS/MS	MCNTs@TpPa-1	tap water	20	0.8-1.5	85.0-106.0	［^24^］
SPME-LC-MS	aptamer@AuNPs@UiO-66-NH_2_	lake water，tap water	5	4	92.0-96.8	［^37^］
d-SPE-HPLC-MS	HFH-COF	biological samples	20	0.6-0.8	85.7-108.7	［^38^］
SPE-LC-MS	Au-PPy nanocomposite sorbent	tap water	2	1.5	94.1-103.2	［^39^］
CME-LC-MS/MS	HKUST-1@poly（DVB-co-AM-co-GMA）	lake water，tap water，reservoir water	1	0.28-0.60	82.2-112.0	this work

DS-MMIP： double-sided magnetic molecularly imprinted polymer； MCNTs： magnetic carbon nanotube； HFH-COF： hollow flower-like covalent organic framework.

## 3 结论

本研究通过离子液体介导的离子热合成法“一锅”快速制备了HKUST-1@poly（DVB-co-AM-co-GMA）功能化聚合物整体柱，通过引入具有多孔结构和丰富金属活性位点的HKUST-1，有效提高了整体柱的比表面积，增加了材料表面的吸附位点。功能化整体柱基于结构中氨基与MCs之间的氢键作用力、苯环之间的*π-π*相互作用以及铜离子的配位作用有效吸附目标物，显著增强富集效果。建立了HKUST-1@poly（DVB-co-AM-co-GMA）功能化聚合物整体柱的CME方法，并与LC-MS/MS技术联用，对环境水样中3种典型的微囊藻毒素进行了同时离线萃取和分析检测。结果充分证明掺杂HKUST-1可以显著提升聚合物整体柱对MCs的富集性能，所建立的联用分析方法展现出极低的检出限、良好的重现性与稳定性，对环境中痕量MCs的监测以及样品预处理小型化研究具有应用价值。
